# Excitatory actions of GABA in the intact neonatal rodent hippocampus *in vitro*

**DOI:** 10.3389/fncel.2013.00020

**Published:** 2013-03-05

**Authors:** Guzel Valeeva, Fliza Valiullina, Roustem Khazipov

**Affiliations:** ^1^Institut de Neurobiologie de la Méditerranée, INSERM U901Marseille, France; ^2^Aix-Marseille UniversityMarseille, France; ^3^Laboratory of Neurobiology, Kazan Federal UniversityKazan, Russia

**Keywords:** GABA, development, hippocampus, giant depolarizing potentials, intracellular chloride, NKCC1, inhibitory post-synaptic potentials

## Abstract

The excitatory action of gamma-aminobutyric acid (GABA) is considered to be a hallmark of the developing nervous system. However, in immature brain slices, excitatory GABA actions may be secondary to neuronal injury during slice preparation. Here, we explored GABA actions in the rodent intact hippocampal preparations and at different depths of hippocampal slices during the early post-natal period [post-natal days (P) 1–7]. We found that in the intact hippocampus at P1–3: (i) GABA exerts depolarizing action as seen in cell-attached single GABA(A) channel recordings; (ii) GABA(A) receptor (GABA(A)-R) agonist isoguvacine and synaptic activation of the GABA(A)-Rs increase the frequency of multiple unit activity and the frequency of the network-driven giant depolarizing potentials (GDPs); and that (iii) Na^+^–K^+^–2Cl^-^ cotransporter (NKCC1) antagonist bumetanide suppresses GDPs and the excitatory actions of isoguvacine. In the hippocampal slices at P2–5, isoguvacine and synaptic activation of GABA(A)-Rs-evoked excitatory responses at all slice depths, including surface and core. Thus, GABA exerts excitatory actions in the intact hippocampus (P1–3) and at all depths of hippocampal slices (P2–5). Therefore, the excitatory actions of GABA in hippocampal slices during the first post-natal days are not due to neuronal injury during slice preparation, and the trauma-related excitatory GABA actions at the slice surface are a fundamentally different phenomenon observed during the second post-natal week.

## INTRODUCTION

Gamma-aminobutyric acid (GABA) is the main inhibitory neurotransmitter in the central nervous system (Freund and Buzsaki, 1996; Farrant and Kaila, 2007). However, early in development, including fetal stages and the first post-natal week in rodents, GABA, acting via GABA(A) receptors (GABA(A)-Rs) often exerts excitatory actions on immature cortical neurons (Cherubini et al., 1991, 2011; Owens and Kriegstein, 2002; Ben-Ari et al., 2007). These excitatory effects of GABA are due to an elevated intracellular chloride concentration and depolarized values of the reversal potential of the GABA(A)-R-activated responses (Obata et al., 1978; Ben-Ari et al., 1989; Owens et al., 1996; Tyzio et al., 2006, 2007, 2008). Elevated chloride in immature neurons is thought to be due to delayed expression of the chloride extruder K^+^–Cl^-^ cotransporter (KCC2) and high expression of the chloride loader Na^+^–K^+^–2Cl^-^ cotransporter (NKCC1) in immature neurons (Kakazu et al., 1999; Rivera et al., 1999; Payne et al., 2003; Yamada et al., 2004; Dzhala et al., 2005; Achilles et al., 2007). Excitatory GABA is essential for the generation of a primitive pattern of network-driven giant depolarizing potentials (GDPs) and controls wide spectrum of developmental phenomena including cell differentiation, migration, and synaptic plasticity (Cherubini et al., 1991, 2011; Owens and Kriegstein, 2002; Khazipov et al., 2004; Sipila et al., 2005; Heck et al., 2006; Ben-Ari et al., 2007). However, excitatory GABA action is not limited to the immature state. A seminal study by van den Pol et al. (1996) revealed that neurons may acquire a secondary excitatory GABA phenotype following various types of trauma including neurites transection. Acquisition of a secondary depolarizing/excitatory GABA phenotype has been confirmed in various models of neuronal trauma and in a number of other pathological conditions including hypoxia, epilepsy, and pain (Cohen et al., 2002; Nabekura et al., 2002; Rivera et al., 2002; Khalilov et al., 2003, 2005; Toyoda et al., 2003; Pond et al., 2006; De Koninck, 2007; Huberfeld et al., 2007; Price et al., 2009; Dzhala et al., 2010).

Are the excitatory actions of GABA during development related to the post-traumatic changes in GABA actions? It should be emphasized that existing evidence for depolarizing and excitatory action of GABA on the immature neurons has been primarily obtained using preparations of brain slices, dissociated neurons or a few days *in vitro* neuronal cultures. Yet, neurons are severely injured during slice preparation, in particular those of them located close to the slice surface (Bak et al., 1980; Khalilov et al., 1997a; Dzhala et al., 2012). Neurons are even more injured during dissociation for acute recordings and neuronal cultures. This raises a hypothesis that excitatory action of GABA on immature tissue actually reflects not a physiological phenomenon but rather a developmental aspect of traumatic injury. Along with this hypothesis, it has been shown that during the second post-natal week, injured neurons at the slice surface display elevated intracellular chloride concentration and excitatory GABA phenotype whereas neurons in the slice core show low-chloride and are inhibited by GABA (Dzhala et al., 2012). Moreover, neurons in the intact hippocampal formation *in vitro* preparation (Khalilov et al., 1997a) at post-natal days (P) 5–7 also display low-chloride values and an inhibitory GABA phenotype (Dzhala et al., 2012). These observations raise a question of whether the depolarizing and excitatory GABA phenotype in immature neurons during the first post-natal week is a primary, developmental phenomenon or it is rather secondary to the shear injury of neurons during slice preparation, a question highly debated recently (Ben-Ari et al., 2012; Bregestovski and Bernard, 2012).

Here, we addressed this question in the intact hippocampal preparation and at different depths of hippocampal slices prepared from both mice and rats during the first post-natal week. We show that GABA exerts depolarizing and excitatory actions during the first post-natal days in both of these preparations and thus it is not a result of neuronal injury during slice preparation – a phenomenon which is observed during the second post-natal week.

## MATERIALS AND METHODS

### ETHICAL APPROVAL

All animal-use protocols conformed to the guidelines of the French National Institute of Health and Medical Research (INSERM) on the use of laboratory animals.

### INTACT HIPPOCAMPUS AND ACUTE HIPPOCAMPAL SLICE PREPARATIONS

Intact hippocampal formations were prepared from P1–7 Swiss mice and Wistar rat pups of both sexes as described earlier (Khalilov et al., 1997a; Khazipov et al., 1999). The animals were either cryoanesthetized (P1–4 animals) or anesthetized with chloral hydrate (P5–7) 350 mg/kg, intraperitoneally. The brain was rapidly removed to oxygenated (95% O_2_–5% CO_2_) ice-cold (2–5°C) artificial cerebrospinal fluid (ACSF) of the following composition (in mM): NaCl 126, KCl 3.5, CaCl_2_ 2, MgCl_2_ 1.3, NaHCO_3_ 25, NaH_2_PO_4_ 1.2, and glucose 11 (pH 7.4). The hemispheres were separated and after removing the cerebellum, the frontal part of the neocortex and surrounding structures, the intact hippocampi were dissected from the septohippocampal complex. The hippocampi were incubated in oxygenated ACSF at room temperature (20–22°C) for 1–8 h before use. For recordings, the hippocampi were placed into a conventional submerged chamber and continuously superfused with oxygenated ACSF at 30°C and at a standard flow rate of 4–6 ml/min, which was increased to 8 ml/min in the experiments with bath isoguvacine application to accelerate wash in and wash out of the drug. The recordings were performed from the middle third of the intact hippocampus.

Acute hippocampal slices were prepared from P2–5 Swiss mice and Wistar rats. About 550 μm-thick transverse slices were cut from the middle third of hippocampus using a Vibratome (VT 1000E; Leica, Nussloch, Germany). Slices were kept in oxygenated ACSF at room temperature (20–22°C) for at least 1 h before use. For recordings slices were placed into a conventional submerged chamber on a plastic net and superfused on both sides with ACSF at 32°C and at a flow rate of 4 ml/min.

### ELECTROPHYSIOLOGICAL RECORDINGS

Local field potentials and multiple unit activity (MUA) were recorded using linear 16-channel extracellular silicone probes (50 μm separation distance between the electrodes; NeuroNexus Technologies, Inc., Ann Arbor, MI, USA) placed vertically in the CA3 pyramidal cell layer. The signals were amplified (×1000, bandpass 0.1 Hz to 4 kHz) using a custom-build amplifier and digitized at 10 kHz using an analog-to-digital converter Digidata 1322/1440 (Axon Instruments, Union City, CA, USA). Synaptic GABA(A)-R-mediated responses were evoked by electrical stimulation (0.1–0.2 ms pulse duration, 40–90 V pulse amplitude, 5–10 s interstimulus interval) via bipolar glass theta electrodes (tip diameter of 10–20 μ, filled with ACSF) in the presence of antagonists of ionotropic glutamate [15 μM CNQX (6-cyano-7-nitroquinoxaline-2,3-dione) and 40 μM D-APV (D-2-amino-5-phosphonovaleric acid)] and GABA(B)-Rs (1 μM CGP55845). MUA responses were monitored using an extracellular electrode located in the CA3 pyramidal cell layer placed at a distance of <0.5 mm from the stimulation electrode as described previously (Valeeva et al., 2010). Patch-clamp recordings were performed using Axopatch 200B (Axon Instruments, Union City, CA, USA). Patch electrodes were made from borosilicate glass capillaries (GC150F-15, Harvard Apparatus, Edenbridge, UK). Pipette solution for cell-attached recordings of single GABA(A) channels contained (in mM): NaCl 120, tetraethylammonium chloride (TEACl) 20, KCl 5, 4-aminopyridine 5, CaCl_2_ 0.1, MgCl_2_ 10, glucose 10, HEPES (4-(2-hydroxyethyl)-1-piperazineethanesulfonic acid)–NaOH 10 buffered to pH 7.2–7.3 and GABA (1–5 μM) added at the day of experiment from 1 mM frozen stock solution. The driving force for GABA(A)-R-mediated currents was determined from the current–voltage relationships of the currents through single GABA(A) channels as described before (Tyzio et al., 2006, 2008). Membrane potential was assessed using cell-attached recordings of single *N*-methyl-D-aspartate (NMDA) channels as described previously (Leinekugel et al., 1997; Tyzio et al., 2003). Patch pipette solution for recordings of single NMDA channels contained nominally Mg^2+^ free ACSF with NMDA (10 μM), glycine (10 μM), and strychnine (1 μM).

### DRUGS

Reagents were purchased from Sigma (Sigma-Aldrich Inc., USA) and Tocris (Tocris Cookson Inc., USA), prepared as stock solutions and stored before use as aliquots in tightly sealed vials at the manufacturers’ recommended temperatures and conditions.

### DATA ANALYSIS

pCLAMP 10.1 (Axon Instruments, USA) and Origin 7.0 (Microcal Software, Northampton, MA, USA) were used for data acquisition and analysis. Group measures are expressed as means ± SE, error bars also indicate SE. Data were assessed for normality using the Shapiro–Wilk test. The statistical significance of differences for normally distributed data was determined with the Student’s *t*-test (paired and unpaired). The Mann–Whitney test and Wilcoxon signed rank test were used to compare non-normally distributed data. Unless indicated otherwise, the level of significance was set at *P* < 0.05.

## RESULTS

### EXCITATORY ACTIONS OF GABA IN THE INTACT HIPPOCAMPUS

**FIGURE 1:**
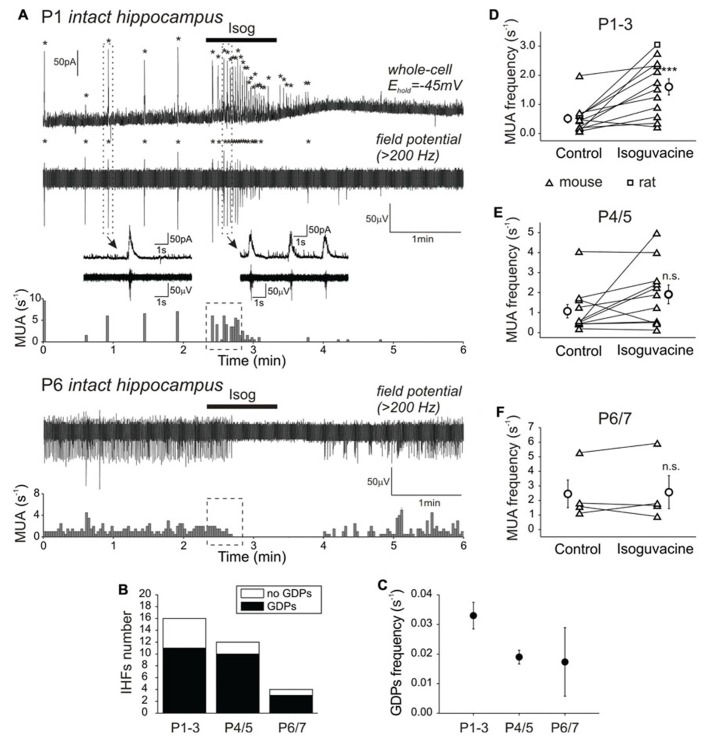
**Effects of the GABA(A) receptor agonist isoguvacine on neuronal activity in the intact *in vitro* hippocampus during the first post-natal week**. **(A)** Example recordings of extracellular MUA responses to isoguvacine application (10 μì, marked with a bar) in a CA3 pyramidal cell layer of P1 and P6 intact mice hippocampi *in vitro*. Concomitant whole-cell voltage clamp recordings from a putative CA3 pyramidal cell are also shown for P1 intact hippocampus above the MUA trace. GDPs are labeled with asterisks. One event before and several events during isoguvacine application are given in the insets on expanded time scale. **(B)** Summary plot of GDPs presence in the intact hippocampi recorded on P1–P7. **(C)** Age-dependence of GDPs frequency in the intact hippocampi during the first post-natal week. **(D–F)** Pooled data on MUA level in control and during isoguvacine application (in the box-outlined area on **A**) in the intact hippocampi of P1–3 **(D)**, P4–5 **(E)**, and P6–7 **(F)** mice (triangles) and rats (squares). Each pair of data points correspond to individual intact hippocampus. Separate circles show mean values with SE.

**FIGURE 2:**
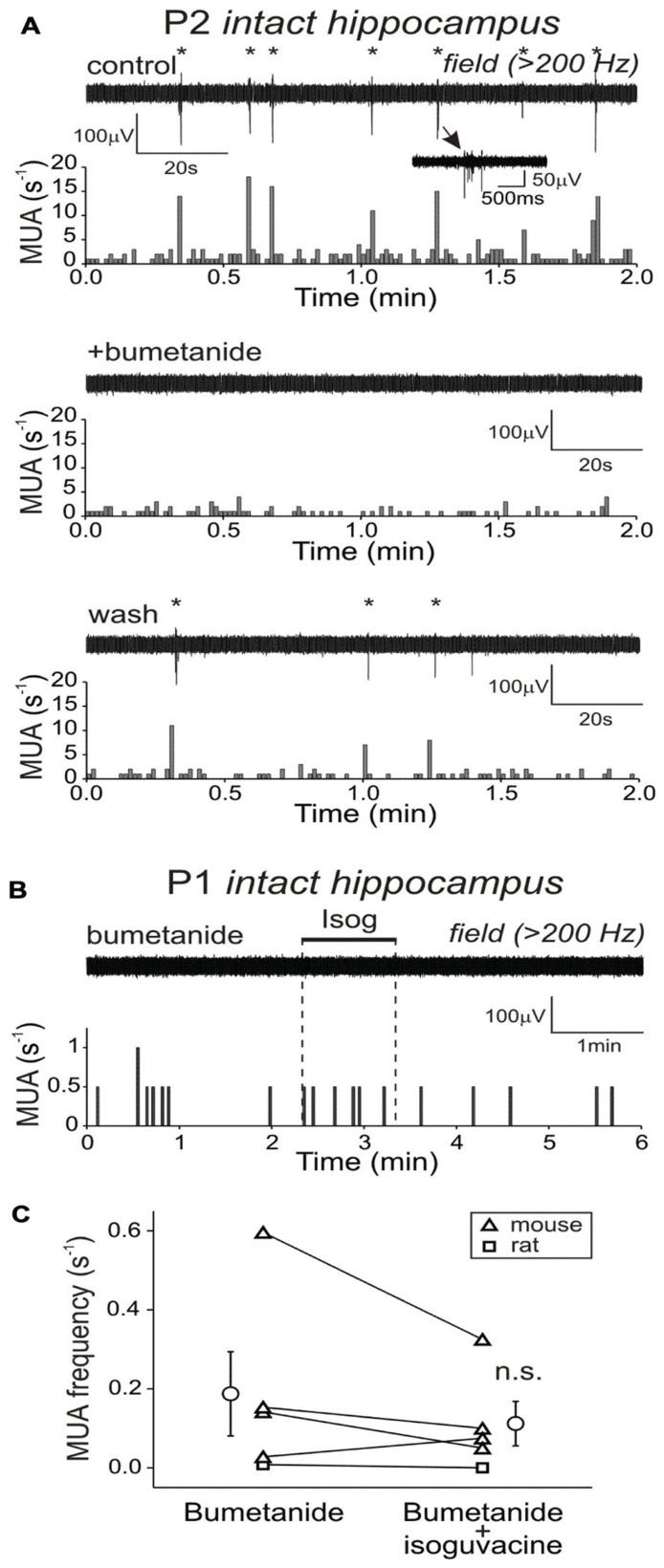
**Na^+^–K^+^–2Cl^–^ co-transporter bumetanide blocks GDPs and prevents the excitatory effect of isoguvacine in the P1–3 intact hippocampus**. **(A)** Field potential recordings of spontaneous network activity in P2 intact hippocampus in control, in the presence of 5 μM bumetanide which blocked GDPs, and after washing out. **(B)** Example trace of MUA response on isoguvacine application in the presence of bumetanide (top) and corresponding histogram of MUA versus time (bottom). **(C)** Summary plot of MUA frequency in the presence of bumetanide and during isoguvacine application. Each pair of data points corresponds to individual intact hippocampus, open circles show mean values ± SE.

We first used extracellular recordings of MUA from the CA3 pyramidal cell layer of the intact hippocampi obtained from P1–7 mice and rats. In keeping with previous studies, extracellular activity in the majority of the intact hippocampi was characterized by the recurrent population bursts of MUA accompanied by the polysynaptic largely GABA(A)-R-mediated currents during concomitant whole-cell recordings from CA3 pyramidal cells, so-called GDPs (Khalilov et al., 1997a, 1999; Leinekugel et al., 1998; Dzhala et al., 1999, 2000; Khazipov et al., 1999). GDPs were observed in the majority of the intact hippocampi (*n* = 24 of 32) through the entire first post-natal week and occurred at 0.002–0.05 s^-1^ (**Figures 1B,C**). In P1–3 intact hippocampi, bath-application of the GABA(A)-R agonist isoguvacine (10 μM for 1 min at a perfusion rate of 8–10 ml/min) evoked large transient GABAergic current in the pyramidal cells and a robust transient increase in GDPs (**Figure 1A**). We estimated the effect of isoguvacine on MUA by normalizing multiple unit frequency during 30-s period following the onset of response to the baseline activity (*n* = 27 intact hippocampi from P1 to P7). In P1–3 intact hippocampi which displayed GDPs in control conditions, isoguvacine increased MUA frequency from 0.5 ± 0.1 to 1.4 ± 0.2 s^-1^ (*n* = 8 intact hippocampi; *P* < 0.01), followed by a longer lasting suppression of activity (**Figures 1A,D**). Isoguvacine produced a similar excitatory effect in the P1–3 intact hippocampi in which GDPs were not present in control conditions. Transient MUA elevation in response to isoguvacine was also observed in 7 of 11 intact hippocampi at P4–5 (*n* = 6 mice; *P* > 0.05; **Figure 1E**), but not in >P5 intact hippocampi, in which isoguvacine did not evoke any significant change in MUA frequency within a 30-s period after the drug application and only the late inhibitory phase could be observed (*n* = 2 mice; *P* > 0.05; **Figures 1A,F**). We also investigated the effect of GABA(A)-R activation by isoguvacine in the presence of bumetanide (5 μM), an inhibitor of NKCC1, which maintains high [Cl^-^]_i_ in the immature cortical neurons (Yamada et al., 2004; Dzhala et al., 2005; Sipila et al., 2006; Tyzio et al., 2006). Bumetanide (5 μM) completely suppressed GDPs in all five of five intact hippocampi regardless of age (P1–5), that is in agreement with previous findings (Dzhala et al., 2012; **Figure 2A**). Bumetanide also prevented the excitatory effect of isoguvacine in P1–3 intact hippocampi, where MUA frequency in the presence of bumetanide and during isoguvacine application averaged 0.19 ± 0.1 and 0.11 ± 0.4 s^-1^, respectively (*n* = 5 intact hippocampi; *P* > 0.05; **Figures 2B,C**).

**FIGURE 3:**
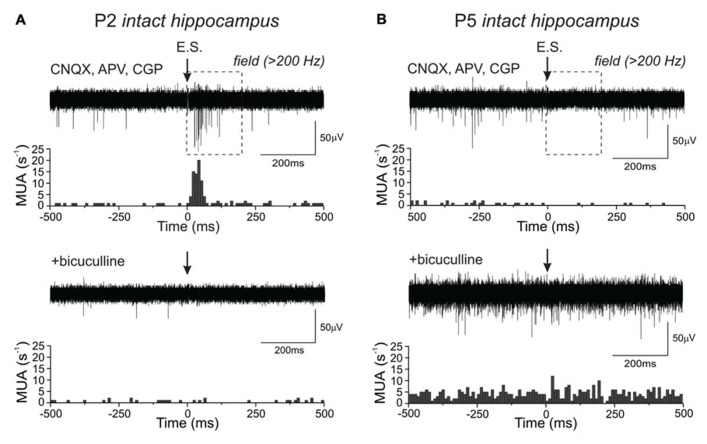
**Age-dependence of the effect of synaptic GABA(A) receptors activation on MUA frequency in the intact hippocampus *in vitro***. **(A,B)** Extracellular recordings of MUA responses evoked by electrical stimulation (E.S.) in the presence of ionotropic glutamate and GABA(B) receptor antagonists (CNQX, APV, CGP55845) in CA3 pyramidal cell layer in P2 **(A)** and P5 **(B)** intact hippocampi; each example shows the superposition of 20 sweeps. The time window in which MUA response was calculated is outlined by the dashed line. Corresponding peristimulus time histograms of spikes normalized to 100 sweeps are shown below each trace. Both excitatory (P2) and inhibitory (P5) responses were completely suppressed by the GABA(A) receptor antagonist bicuculline (bottom).

We next explored the effect of synaptic activation of GABA(A)-Rs on MUA frequency in the intact hippocampi at different post-natal ages (**Figure 3**). GABA(A)-R-mediated post-synaptic responses were evoked by electrical stimulation of the intact hippocampi via bipolar glass theta electrodes in the presence of antagonists of ionotropic glutamate (15 μM CNQX and 40 μM D-APV) and GABA(B)-Rs (1 μM CGP55845). MUA responses were monitored using an extracellular electrode located in the CA3 pyramidal cell layer placed at a distance of <0.5 mm from the stimulation electrode. We have previously shown that under these conditions, stimulation of GABAergic synapses causes an increase in MUA frequency within a time window of the GABA(A)-R-mediated post-synaptic potentials (PSPs) in the rat hippocampal slices until P8 and an inhibition of MUA in >P8 rat hippocampal slices (Tyzio et al., 2008; Valeeva et al., 2010). We found that in the intact hippocampi prepared from P2–3 animals, electrical activation of GABAergic synapses evokes a 13 ± 2-fold increase in MUA frequency within 200 ms time window following the stimulus (*n* = 3; *P* < 0.05; **Figure 3A**), and the effect is suppressed by the GABA(A)-R antagonist bicuculline (20 μM). In the intact hippocampi from P4–5 animals, synaptic activation of GABA(A)-Rs-evoked inhibition of MUA (reduction of MUA frequency to 0.3 ± 0.01 of the baseline activity; *n* = 3 mice; *P* < 0.05; **Figure 3B**), and this inhibitory effect was also suppressed by bicuculline. Thus, activation of the GABA(A)-Rs with exogenous GABA(A) agonist and via the activation of synaptic GABA(A)-Rs results in the excitation of neurons in the intact hippocampi preparation during the first post-natal days, and GABA shifts from excitatory to inhibitory by the end of the first post-natal week. In keeping with previous observations made in hippocampal slices (Khazipov et al., 2004; Tyzio et al., 2007, 2008) the developmental fade of excitatory actions of synaptic GABA(A)-mediated responses occurred prior to the change in the effect of the bath-applied agonist.

### DEPOLARIZING ACTION OF GABA IN THE INTACT HIPPOCAMPI

**FIGURE 4:**
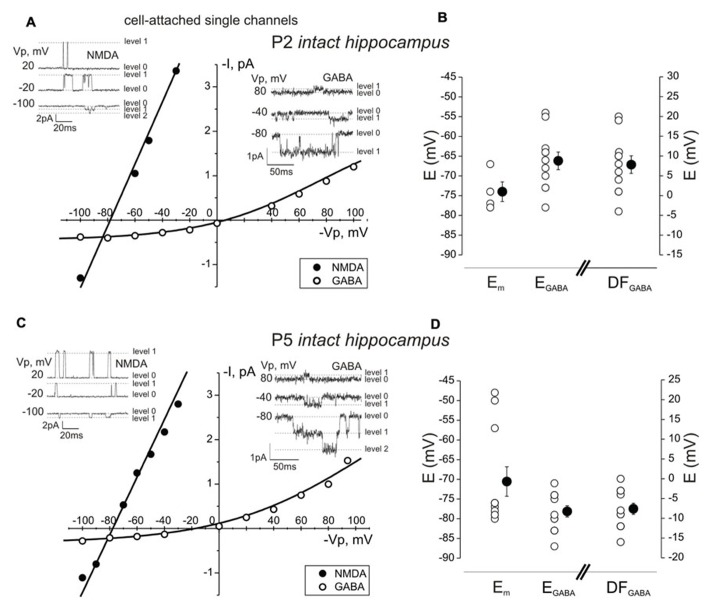
**Age-dependence of the GABA driving force and resting membrane potential in CA3 pyramidal cell layer neurons of the intact hippocampus**. **(A,C)** Measurements of the GABA driving force (DF_GABA_) and membrane potential (*E*_m_) using cell-attached recording of GABA(A) and NMDA channels in the CA3 pyramidal cell layer neurons in P2 **(A)** and P5 **(C)** rat intact hippocampi. Recordings of single channel openings (levels are indicated with dashed lines: level 0 corresponds to closed state, level 1 – to the openings of single channels and level 2 – to the openings of two channels) at different pipette potentials (*V*_p_) are shown in the insets. On the I–V curve for the currents via GABA(A) channels, the reversal potential reflects the GABA driving force value. On the I–V curve for the currents via NMDA channels, the reversal potential reflects the resting membrane potential. **(B,D)** Pooled data on resting membrane potential (*E*_m_), the reversal potential of GABA-activated currents (*E*_GABA_, calculated for each cell as mean *E*_m_ + DF_GABA_) and DF_GABA_ in P2 **(B)** and P5 **(D)** intact hippocampi. Each open circle corresponds to an individual neuron, closed circles show mean values ± SE.

The excitatory action of GABA on immature neurons is due to the depolarized values of the GABA(A) reversal potential compared to the resting membrane potential and therefore, to the depolarizing driving force acting on currents through the GABA(A) channels (DF_GABA_). To measure DF_GABA_ in the intact hippocampal neurons, we used non-invasive cell-attached recordings of single GABA(A) channels that do not perturb intracellular ionic content (Tyzio et al., 2006, 2008). Examples of single channel recordings at different pipette potentials (*V*_p_) and the resulting current–voltage relationships in the intact hippocampi from P2 and P5 rats are shown in **Figure 4**. DF_GABA_ was deduced from the I–V curves as a reversal potential of the currents through the GABA channels. At P2, neurons had values of DF_GABA_ in the range from -4 to 20 mV (mean, 8 ± 2 mV; *n* = 11 cells; **Figures 4A,B**). We next measured the resting membrane potential (*E*_m_) of cells in the CA3 pyramidal cell layer from the reversal potential of the currents through single NMDA channels recorded in cell-attached mode (Leinekugel et al., 1997; Tyzio et al., 2008). This approach was employed to avoid an introduction of the shunting conductance and neuronal depolarization occurring in whole-cell recordings from small high input resistance neurons (Tyzio et al., 2003, 2008). Deduced from the reversal potential of the currents through NMDA channels values of *E*_m_ in P2 intact hippocampi ranged from -78 to -67 mV (mean *E*_m_, -74 ± 2 mV; *n* = 4 cells; **Figures 4A,B**). These values were in the range of the values previously reported in hippocampal slices (Tyzio et al., 2003, 2008). In agreement with previous findings (Tyzio et al., 2003), the membrane potential in whole-cell recordings was -53 ± 1 mV (*n* = 4). Knowing *E*_m_ and DF_GABA_ we further estimated the reversal potential of the GABA(A)-activated conductance (*E*_GABA_ = mean*E*_m_ - DF_GABA_). In P2 intact hippocampal neurons, *E*_GABA_ was in the range from -78 to -54 mV (mean, -66 ± 2 mV; *n* = 11 cells; **Figure 4B**) which is close to *E*_GABA_ estimates using a similar approach in hippocampal slices (-63 mV; Tyzio et al., 2008). Similar measurements performed in P5 intact hippocampi revealed *E*_m_ values of -71 ± 4 mV (*n* = 11 cells; *P* > 0.05 for comparison with *E*_m_ at P2), and slightly hyperpolarizing DF_GABA_ values (-8 ± 1 mV; *n* = 12 cells; *P* < 0.001 compared to P2). As a result, *E*_GABA_ values at P5 were more negative than at P2 (-78 ± 1 mV; *P* < 0.001 compared to P2). This differs from slices obtained from P5 rats in which GABA remains depolarizing (by +11 mV) and *E*_GABA_ values are more positive (-64 mV) than in the intact hippocampi (Tyzio et al., 2008). Taking into account the values of *E*_GABA_ we calculated intracellular chloride concentrations at P2 and P5 of 10.5 ± 0.9 and 6.9 ± 0.3 mM, respectively. Thus, neurons in the intact hippocampi are depolarized by GABA at P2 but are hyperpolarized at P5. These findings are consistent with the developmental changes in the effects of synaptic GABA on MUA seen with extracellular recordings, which revealed excitatory and inhibitory GABA actions in P1–3 and P4–5 intact hippocampi, respectively.

### DEPTH-PROFILE OF THE GABA ACTIONS IN HIPPOCAMPAL SLICES

**FIGURE 5:**
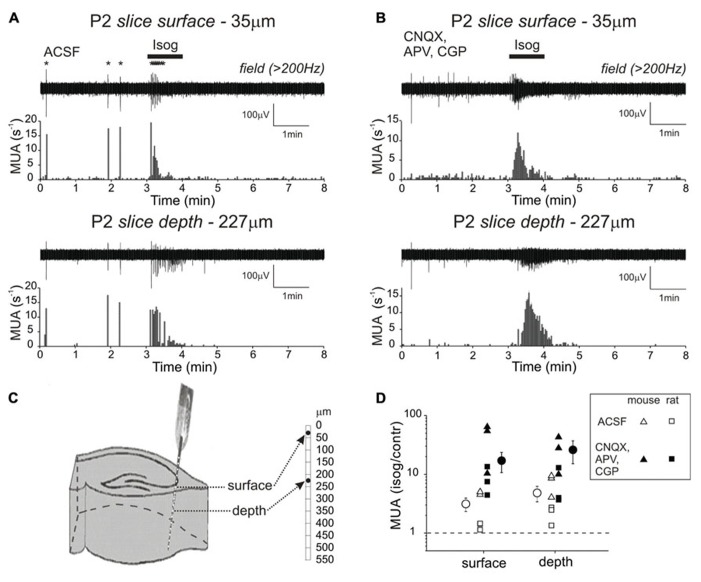
**Depth-dependence of isoguvacine effects on MUA in the hippocampal slice at P2–4**. Scheme of recording setup is shown on **(C)**. A 16-channel silicone probe (50 μm distance between the electrodes) is inserted vertically in the CA3 pyramidal cell layer of 550 μm-thick slice. Recordings from the electrodes indicated as “depth” and “surface” are shown on **(A,B)**. **(A,B)** Extracellular recordings of MUA responses evoked by bath-applied GABA(A) receptor agonist isoguvacine from the surface and deep electrodes of the 16 site silicone probe in control conditions **(A)** and in the presence of glutamate and GABA(B) receptor antagonists (CNQX, APV, CGP55845) **(B)**. **(D)** Summary plot of MUA response on isoguvacine application, normalized to control level of activity in ACSF (open symbols) and in the presence of CNQX, APV, CGP55845 (closed symbols). Each data point corresponds to an individual intact hippocampus. Mean values are shown by the circles with error bars indicating SE.

It has been previously shown that in hippocampal slices during the second post-natal week, GABA exerts excitatory action on neurons located close to the slice surface whereas the neurons located in the core of slice are inhibited by GABA (Dzhala et al., 2012). The depth-dependence in the action of GABA coincided with the neuronal damage which was most prominent at the slice surface. Therefore we explored whether the actions of GABA differ at different depths of 550 μm-thick slices during the first post-natal week using linear 16 channel silicone probes (50 μm distance between the electrodes) inserted vertically into the slice to monitor MUA in the CA3 pyramidal cell layer throughout the entire slice depth (**Figure 5C**). In control conditions, MUA was organized in GDPs synchronously generated at all depths of P2–4 hippocampal slices. GDPs were observed in each slice studied (*n* = 6) and their occurrence rate of 0.08 ± 0.04 s^-1^ was higher than that of GDPs in the intact hippocampi.

Giant depolarizing potentials typically co-occurred at all depths, often synchronously but also as propagating waves. A variety of across-slice propagation patterns has been observed with leading electrodes placed either at the surface or within the slice. However, cross-correlation analysis of MUA during GDPs recorded from different depths indicated that neuronal excitation is synchronized through the entire depth in a time window of 100–300 ms without any systematic propagation gradient. In none of the experiments could we observe an inhibition of MUA in the core of the slice during surface GDPs. Bath-application of isoguvacine (10 μM for 1 min) evoked a transient increase in MUA and GDPs frequency at all depths (**Figures 5A,B**), and the level of MUA elevation was similar in the slice core and surface (from 2 ± 0.9 to 6 ± 2 s^-1^, and from 0.6 ± 0.1 to 2 ± 0.6 s^-1^, respectively; *n* = 6 slices; *P* < 0.05; P2–4). Similar excitation at all depths in response to isoguvacine was also observed in P7–11 slices (from 2 ± 0.4 to 5 ± 1 s^-1^ in the surface, and from 5 ± 1 to 14 ± 2 s^-1^ in the depth of slice; *n* = 14; *P* < 0.001). In the presence of the ionotropic glutamate receptor antagonists CNQX and APV, GDPs were completely abolished and the overall level of MUA frequency was strongly reduced. However, application of isoguvacine evoked a uniform increase in MUA frequency at all slice depths at P2–4 (**Figures 5B,D**; note that the excitatory effects appear to be stronger in mice compared to rats). This differs from a previously reported sandwich-like structure of MUA response to isoguvacine at P7–14, composed of excitation at surfaces (which is bumetanide-insensitive) and inhibition in the core of slice observed after blockade of glutamate receptors (Dzhala et al., 2012). Thus, excitation through all slice depth was observed at P2–4 independently on whether glutamate receptors are blocked or not, whereas in ≥P7 slices excitation in the core of slice was seen only when glutamate receptors are not blocked. Likely explanation for this difference is that in ≥P7 slices GABA(A)-R-mediated excitation of damaged pyramidal cells at the slice surface causes excitation of cells deep in slice via local glutamatergic synapses, and this excitation overrides isoguvacine-mediated inhibition of the deep cells. 

**FIGURE 6:**
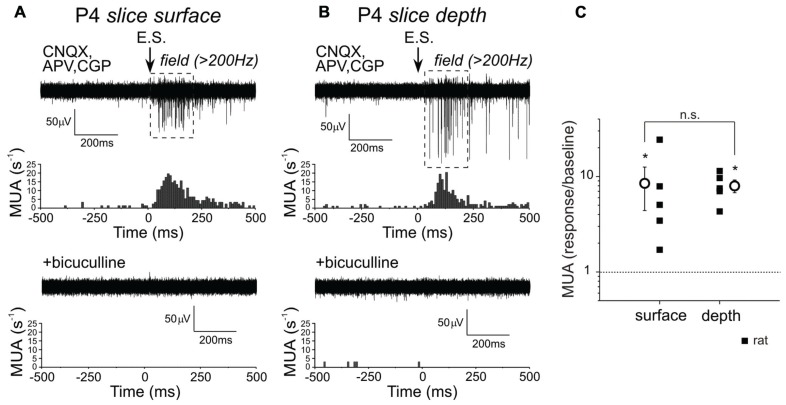
**Depth-dependence of MUA response on synaptic GABA(A) receptor activation in P4–5 rat hippocampal slices**. **(A,B)** Example field potential recording of MUA response evoked by electrical stimulation (E.S.) at the surface **(A)** and in the core **(B)** of a P4 hippocampal slice in the presence of ionotropic glutamate and GABA(B) receptor antagonists (CNQX, APV, CGP55845). Suppression of MUA response by 20 μM bicuculline is shown at the bottom. Corresponding peristimulus spike time histograms normalized to 100 sweeps are shown below each trace. **(C)** Summary plot of electrically evoked MUA response, normalized to the baseline at the surface and depth of the slice. Squares reflect the normalized MUA response at the depth and the surface of an individual slice, circles show mean values ± SE.

Using a similar recording setup, we also explored the effects of synaptic activation of GABA(A)-Rs on MUA at different depth in slices from P4–5 rats, at the time when synaptic GABA responses in the intact hippocampi are already inhibitory (**Figure 6**). Pharmacologically isolated GABA(A)-R-mediated PSPs caused an increase in CA3 pyramidal cell layer MUA frequency within a time window of about 200 ms both in the slice core and surface (from 1 ± 0.4 to 8 ± 3 s^-1^ and from 1 ± 0.6 to 9 ± 3 s^-1^ correspondingly; *n* = 5; *P* < 0.05). The effects were completely suppressed by the blockade of GABA(A)-Rs with bicuculline. Thus, activation of GABA(A)-Rs with exogenous GABA(A) agonist and by direct stimulation of interneurons results in the excitation of neurons both in the core and at the surface of slice during the first post-natal week suggesting that the GABAergic excitation observed in slices does not result from the injury of neurons at the slice surface. This is in contrast to the findings obtained in slices during the second post-natal week, where GABAergic excitation is limited to the slice surface whereas neurons located in the core of slices are inhibited by GABA (Dzhala et al., 2012).

## DISCUSSION

The principal findings of the present study can be summarized as the following: (i) excitatory actions of GABA were revealed in intact rodent (rat and mice) hippocampi in response to the exogenous GABA(A)-R agonist isoguvacine and synaptic activation of GABA(A)-Rs using non-invasive extracellular recordings of MUA during the P1–3; these excitatory GABA actions faded by P4–5; (ii) the depolarizing action of GABA on hippocampal neurons was revealed using non-invasive cell-attached recordings of single GABA(A) channels in the intact hippocampi at P2 and hyperpolarizing GABA actions were observed in the intact hippocampi at P5; (iii) the excitatory actions of GABA and GDPs in immature intact hippocampi were suppressed by the NKCC1 antagonist bumetanide. Finally, (iv) the excitatory actions of GABA were revealed at all depths of P2–5 hippocampal slices in response to exogenous GABA(A)-R agonist and to the synaptic activation of GABA(A)-Rs, and spontaneous GDPs were also observed at all depths of slices. Together with previous findings, these results indicate that the excitatory actions of GABA on hippocampal neurons during development involve two age-specific mechanisms: (i) developmental, which is present both in the intact hippocampi and in slices during the first post-natal days, and which fades by the end of the first post-natal week, and (ii) related to injury elevation of intracellular chloride associated with an acquisition of a secondary excitatory GABA phenotype, which is primarily expressed at the slice surface during the second post-natal week.

This study presents evidence that GABA exerts depolarizing and excitatory actions in the intact hippocampi during the first post-natal days (P1–3), and through all depths of hippocampal slices during the first post-natal week. So far, actions of the bath-applied GABA(A)-R agonist on MUA in the intact hippocampi were only previously described at P5–7, and these were inhibitory (Dzhala et al., 2010, 2012). This has led to a hypothesis that depolarizing and excitatory actions of GABA during development are an experimental artifact of slice preparation (Bregestovski and Bernard, 2012). In the present study, we show that during the first post-natal days bath-applied isoguvacine and synaptic activation of GABA(A)-Rs augments MUA in the intact hippocampi as seen with non-invasive extracellular recordings, and that GABA depolarizes immature neurons using cell-attached recordings of single GABA(A) channels. We also found that bath-applied isoguvacine and synaptic activation of GABA(A)-Rs evoke neuronal excitation at all depths of slices during the first post-natal week. Similar depolarizing developmental GABA actions have been also observed in several other preparations maintaining the intact neural structure *in vitro* such as preparations of immature retina from various species including the embryonic chick (Yamashita and Fukuda, 1993), ferret (Fischer et al., 1998), turtle (Sernagor et al., 2003), and rabbit (Zheng et al., 2004), developing optic tectum of *Xenopus laevis* tadpoles isolated brain *in vitro* (Khakhalin and Aizenman, 2012), Cajal–Retzius cells in tangential slices (Kolbaev et al., 2011a,b) and rat spinal cord *in vitro* (Nishimaru et al., 1996; Fellippa-Marques et al., 2000; Bos et al., 2011). Altogether, these findings show that GABA is excitatory in the immature brain both in the intact preparations and slices *in vitro* and disprove a hypothesis that the early excitatory GABA actions are secondary to neuronal trauma during slice preparation.

It should be emphasized that while GABA exerts depolarizing and excitatory actions in the immature intact hippocampus, it may also exert inhibitory actions, however. Indeed, blockade of GABA(A)-Rs transforms GDPs to hypersynchronous epileptiform discharges: interictal-like activity starting from P0 and ictal-like activity starting from P2 in the intact hippocampus *in vitro* (Khalilov et al., 1997a,b, 1999). Similar epileptiform transformation after blockade of GABA(A)-Rs are also observed in slices: interictal-like activity in the hippocampal slices is seen starting from P0 (Khazipov et al., 1997; Khalilov et al., 1999; Lamsa et al., 2000; Wells et al., 2000) and seizure-like events in the slices at P9–19 (Swann and Brady, 1984; Gomez-Di et al., 1997). Blockade of GABA(A)-Rs also was shown to induce electrographic and behavioral seizures in the rat pups *in vivo* already from P3 (Baram and Snead, 1990). The allosteric modulators of the GABA(A)-Rs (barbiturates, benzodiazepines) are efficient in suppressing early life seizures in several animal models *in vivo* (Smythe et al., 1988; Kubova and Mares, 1991; Velisek et al., 1995; Kubova et al., 1999; Isaev et al., 2005) and desynchronize neuronal firing and slow down propagation of GDPs in hippocampal slices during the first post-natal week (Valeeva et al., 2010). Desynchronization of neuronal firing by GABA is in part due to slow and variable action potential delays at the excitatory GABAergic synapses, caused by relatively small levels of neuronal depolarization during GABAergic post-synaptic responses that do not attain the action potential threshold and therefore activation of a non-inactivating sodium conductance (at about -60 mV) is required to trigger action potentials (Sipila et al., 2005; Valeeva et al., 2010). Additional factors are shunting of excitatory glutamatergic inputs due to an increase in membrane conductance as a result of opening of GABA(A) channels and voltage-gated potassium channels, and inactivation of sodium channels (Staley and Mody, 1992; Gao et al., 1998; Lu and Trussell, 2001).

While no major difference in the action of GABA was seen in slices and in the intact hippocampi at P1–3, differences between these two preparations were observed at P4–5, when GABA exerted excitatory actions in slices but either inhibited (inhibition of MUA in response to stimulation of GABAergic synapses, hyperpolarizing GABA seen with cell-attached recordings of GABA channels) or had no significant effect (bath-applied isoguvacine) in the intact hippocampi. Deduced from cell-attached recordings values of intracellular chloride according to the Nernst equation were fairly low in P5 intact hippocampi (6.9 ± 0.3 mM). Furthermore, these estimates drop to about 4 mM of intracellular chloride after correction for bicarbonate conductance [which equals about 3 mM of chloride, given that intracellular pH of about 7.2 would provide a bicarbonate concentration (15 mM) that equals about 0.2 × 15 = 3 mM of chloride, where 0.2 is the approximate bicarbonate permeability of GABA(A)-Rs]. Interestingly, these electrophysiological estimates of the intracellular chloride (4 mM after correction for bicarbonate conductance) are somewhat lower than those (10–17 mM, depending on depth) obtained using chlomeleon imaging in the intact hippocampi of P5–7 mice (Dzhala et al., 2012). An important note should be made, however, that our cell-attached estimates of the intracellular chloride have been likely made for the soma and these can be higher in other compartments such as the axon initial hillock (Szabadics et al., 2006; Khirug et al., 2008). It should also be added that the values of *E*_GABA_ are heterogeneous and that our measures of this parameter as well as measures of GABA actions (with bath-applied isoguvacine and synaptic GABA) on MUA only give an estimate of a general trend in the developmental changes in GABA actions. It is conceivable that the population response to GABA is composed of both inhibitory and excitatory responses and the “net” change only reflects a summation of these two. If only a minority of cells is excited by GABA in the population, the net effect would be inhibitory. This minority is likely to be essential for driving population activity, however, as it has been demonstrated for the generation of interictal events in the epileptic slices of subiculum in temporal lobe epilepsy patients where only small proportion of cells were depolarized by GABA but these were efficient to drive interictal events (Cohen et al., 2002). This could also explain an apparent contradiction in our findings: while no net excitatory GABA actions were revealed in P4–5 intact hippocampus, bumetanide-sensitive GDPs were still present in this preparation (although at reduced frequency) and those could be driven by the minority of neurons with excitatory GABA which were not sampled in our cell-attached recordings and whose activation during GABAergic stimulation was obscured by inhibitory response in larger neuronal population with extracellular MUA recordings.

Yet, it remains that the net effects of GABA at P4–5 fundamentally differ in slices (excitation) and intact hippocampus preparation (inhibition). What are the reasons for this discrepancy? This may involve acidosis developing in the intact hippocampi obtained from older animals in this relatively thick preparation and pH measurements indeed indicate an acidosis developing in ≥P4 intact hippocampus core (see Voipio and Kaila, 1993; K. Kaila, personal communication). Under acidosis, a negative shift in *E*_GABA_ resulting from AE3 Cl/HCO_3_ exchanger activation is conceivable (Pfeffer et al., 2009). Acidosis also reduces the frequency of GDPs (Ruusuvuori et al., 2010) that could also explain reduced frequency of GDPs in the intact hippocampi compared to that in the hippocampal slices. An alternative yet unlikely explanation could be that elevated chloride is caused by traumatic injury to neurons in slices as described previously using slices obtained during the second post-natal week (Dzhala et al., 2012). However, an excitatory GABA phenotype was observed in P4–5 slices at all slice depths, whereas the characteristic for secondary trauma-related excitatory GABA phenotype observed during the second post-natal week is characterized by a sandwich-like structure, with an excitation at the surface and inhibition at depth (Dzhala et al., 2012). Therefore, trauma-related excitatory GABA phenotype may contribute, but it is clearly not the principal cause of excitatory GABA observed at the end of the first post-natal week in slices.

Despite of a seeming contradiction, there is no discrepancy between this and previous work by Dzhala et al. (2012). It appears that all differences in GABA actions can be explained simply on the basis of the difference in the developmental stage of the hippocampus in these two studies, where the present one focuses on the first post-natal week. During this period including ages P1–3 in the intact hippocampus preparation and P1–6 slices (and also likely during the fetal stage) GABA is excitatory regardless of whether brain is damaged or not, and GDPs observed in slices are not secondary to the trauma during slicing. The phenomenon of secondary trauma-related excitatory GABA action is observed and is limited to the neurons at the slice surface during the second post-natal week. Thus, excitatory actions of GABA on hippocampal neurons during development involve two different age-specific mechanisms: (i) developmental, which is present both in the intact hippocampi and in slices (though all depths) during the first post-natal days, and which fades by the end of the first post-natal week, and (ii) related to an injury induced elevation of intracellular chloride associated with an acquisition of a secondary excitatory GABA phenotype, which is primarily expressed at the slice surface during the second post-natal week.

## Conflict of Interest Statement

The authors declare that the research was conducted in the absence of any commercial or financial relationships that could be construed as a potential conflict of interest.

## AUTHOR CONTRIBUTIONS

Guzel Valeeva and Roustem Khazipov conceived the project and designed experiments. Guzel Valeeva and Fliza Valiullina performed the experiments. Guzel Valeeva analyzed the data. Roustem Khazipov and Guzel Valeeva wrote the paper.
